# Biglycan: A Multivalent Proteoglycan Providing Structure and Signals

**DOI:** 10.1369/0022155412456380

**Published:** 2012-12

**Authors:** Madalina V. Nastase, Marian F. Young, Liliana Schaefer

**Affiliations:** Pharmazentrum Frankfurt/ZAFES, Institut für Allgemeine Pharmakologie und Toxikologie, Klinikum der Goethe-Universität Frankfurt am Main, Frankfurt am Main, Germany (MVN,LS); Craniofacial and Skeletal Diseases Branch, National Institute of Dental Research, National Institutes of Health, Bethesda, Maryland (MFY)

**Keywords:** extracellular matrix, proteoglycan, Toll-like receptor, inflammasome, TGFβ, inflammation, bone, skeletal muscle, Duchenne’s muscular dystrophy, sepsis, Wnt

## Abstract

Research over the past few years has provided fascinating results indicating that biglycan, besides being a ubiquitous structural component of the extracellular matrix (ECM), may act as a signaling molecule. Proteolytically released from the ECM, biglycan acts as a danger signal signifying tissue stress or injury. As a ligand of innate immunity receptors and activator of the inflammasome, biglycan stimulates multifunctional proinflammatory signaling linking the innate to the adaptive immune response. By clustering several types of receptors on the cell surface and orchestrating their downstream signaling events, biglycan is capable to autonomously trigger sterile inflammation and to potentiate the inflammatory response to microbial invasion. Besides operating in a broad biological context, biglycan also displays tissue-specific affinities to certain receptors and structural components, thereby playing a crucial role in bone formation, muscle integrity, and synapse stability at the neuromuscular junction. This review attempts to provide a concise summary of recent data regarding the involvement of biglycan in the regulation of inflammation and the musculoskeletal system, pointing out both a signaling and a structural role for this proteoglycan. The potential of biglycan as a novel therapeutic target or agent for the treatment of inflammatory diseases and skeletal muscular dystrophies is also addressed.

Biglycan is a member of the class I family of small leucine-rich proteoglycans (SLRPs) (Schaefer and Iozzo 2008). The biglycan gene has been mapped to the X chromosome (McBride et al. 1990). It encodes for a 42-kDa protein core (Bianco et al. 1990) containing leucine-rich repeats (LRRs), to which one or two glycosaminoglycan (GAG) side chains are covalently bound. The tissue-specific chondroitin- or dermatan-sulfate GAG chains of biglycan are attached to amino acid residues at the N-terminus of the core protein (Choi et al. 1989; Roughley and White 1989). There are some indications that biglycan might be a “part-time” proteoglycan as its non-glycated form has been found in aging articular cartilage and intervertebral discs (Johnstone et al. 1993; Roughley et al. 1993). Detailed structural characteristics of biglycan have been provided in recent reviews (Schaefer and Iozzo 2008; Schaefer and Schaefer 2010).

Biglycan, which is expressed ubiquitously (Bianco et al. 1990; Ungefroren et al. 1998), is synthesized as a precursor from which an N-terminal propeptide is cleaved off by bone morphogenetic protein (BMP) 1 to yield the mature form (Scott et al. 2000). Secreted biglycan interacts via its core protein or GAG chains with numerous components of the extracellular matrix (ECM)—for example, type I, II, III, and VI collagen and elastin (Schonherr et al. 1995; Hunzelmann et al. 1996; Reinboth et al. 2002; Douglas et al. 2006; Hwang et al. 2008)—thereby becoming sequestered in the ECM of most organs.

Since the discovery of biglycan in developing bone almost 30 years ago (Fisher et al. 1983), a multitude of studies have tried to elucidate the biological role of this proteoglycan. Initially, biglycan was considered to be merely a static structural component of the ECM. Osteoporosis-like phenotype and abnormalities of collagen fibrils observed in biglycan-deficient mice initiated a number of investigations addressing the mechanisms of biglycan-dependent regulation of bone formation and collagen fiber assembly (Xu et al. 1998; Ameye et al. 2002; Corsi et al. 2002; Bi et al. 2005; Bi et al. 2007; Zhang et al. 2009; Embree et al. 2010). Its ability to interact with transforming growth factor (TGF) β (Hildebrand et al. 1994); tumor necrosis factor (TNF)-α (Tufvesson and Westergren-Thorsson 2002); BMP2, -4, and -6 (Chen XD et al. 2004; Mochida et al. 2006); and Wnt-1-induced secreted protein 1 (WISP1) (Desnoyers et al. 2001) established this small leucine-rich proteoglycan (SLRP) as a modulator of growth factors and cytokine functions. Recent studies discovering biglycan as a signaling molecule (Schaefer et al. 2005; Babelova et al. 2009; Moreth et al. 2010; Berendsen et al. 2011) and a ligand of Toll-like receptors (TLRs)-2 and -4 (Schaefer et al. 2005), P2X7/P2X4 purinergic receptors (Babelova et al. 2009), low-density lipoprotein receptor-related protein 6 (LRP6) (Berendsen et al. 2011), or receptor tyrosine kinase MuSK (Amenta et al. 2012) gave rise to a new paradigm of how this proteoglycan regulates a host of biological processes. It firmly established biglycan as a part of the innate immune system (Schaefer et al. 2005) and a regulator of osteogenesis (Berendsen et al. 2011; Moreth et al. 2012), synaptic stability (Amenta et al. 2012), and muscle integrity (Mercado et al. 2006; Rafii et al. 2006; Amenta et al. 2011). The observation that biglycan is capable of clustering several types of receptors and orchestrating their signaling (Babelova et al. 2009) further underlines the complexity of the biglycan signaling networks.

Reflecting its widespread expression and complex function, involvement of biglycan in numerous experimental and human diseases has been reported (Table 1). Details have been summarized in recent reviews on the SLRP family (Merline et al. 2009; Iozzo and Schaefer 2010; Kalamajski and Oldberg 2010; Schaefer and Schaefer 2010; Theocharis et al. 2010; Schaefer 2011; Moreth et al. 2012; Schaefer and Iozzo 2012). However, one has to be aware that some of the conclusions on the importance of biglycan in certain diseases are exclusively based on histological findings. New findings indicate that only unsequestered biglycan is capable of acting as a signaling molecule at least in inflammation (Schaefer et al. 2005; Babelova et al. 2009). Therefore, the amount of biglycan in tissue sections does not necessarily reflect its biological effect as it represents mainly biglycan that has been sequestered in the ECM, for example, as part of the fibrotic scar (Schaefer 2011).

**Table 1.** Involvement of Biglycan in Selected Experimental and Human Diseases

Disease	Species	References
Alzheimer disease	Mouse	Lam et al. 2011
Aortic dissection	Mouse, human	Bellucci et al. 2007; Heegaard et al. 2007
Asthma	Mouse, rat, human	Pini et al. 2007; Pinelli et al. 2009; Nihlberg et al. 2010; Venkatesan et al. 2012
Atherosclerosis	Mouse, rat, human	Skalen et al. 2002; Nakashima et al. 2007
Cancer	Human	Mintz et al. 2005; Coulson-Thomas et al. 2011; Gu et al. 2012; Wang B et al. 2011
Diabetes	Mouse, rat, human	Schaefer et al. 2001; Thompson et al. 2011; Bolton et al. 2012
Duchenne muscular dystrophy	Mouse, human	Fadic et al. 2006; Mercado et al. 2006; Brandan et al. 2008; Amenta et al. 2011
Intervertebral disc disorders	Human	Brown et al. 2012
Fibrotic liver disease	Rat, human	Meyer et al. 1992; Krull et al. 1993; Gressner 1994; Hogemann et al. 1997
Myocardial infarction	Mouse, human	Campbell et al. 2008; Westermann et al. 2008
Multiple sclerosis	Human	Mohan et al. 2010
Osteoarthritis	Mouse	Wadhwa et al. 2005; Embree et al. 2010
Osteoporosis	Mouse	Xu et al. 1998
Perimyocarditis	Mouse	Popovic et al. 2011
Fibrotic kidney disease	Mouse, rat, human	Schaefer et al. 1998; Schaefer et al. 2000; Kuroda et al. 2004; Schaefer 2011
Rheumatic arthritis	Human	Polgar et al. 2003; Sokolove et al. 2012; Antipova and Orgel 2012
Sepsis	Mouse	Schaefer et al. 2005; Babelova et al. 2009
Systemic lupus erythematosus	Mouse, human	Moreth et al. 2010

Recent data provide evidence for a crucial role of biglycan in the regulation of inflammation, bone growth, and muscle development and regeneration. This brief review aims to focus on the mechanisms and consequences of biglycan interaction with cell surface molecules/receptors in those processes to underline the role of biglycan as a multivalent, matrix-derived signaling molecule.

## Biglycan Signaling in Inflammation

Research over the past few years has provided strong evidence that biglycan in its soluble form acts as danger signal bridging the innate and adaptive immune systems (Schaefer et al. 2005; Babelova et al. 2009; Moreth et al. 2010; Popovic et al. 2011; Schaefer and Iozzo 2012). Following tissue stress and injury, biglycan is proteolytically released from the extracellular matrix and turns into a host-derived non-microbial danger signal (damage-associated molecular patterns, DAMPs), which is recognized by innate immunity receptors in a manner similar to the function of pathogen-associated molecular patterns (PAMPs) (Chen GY and Nunez 2010). Biglycan core protein can be cleaved by several proteolytic enzymes, such as BMP1, matrix metalloproteinase (MMP)-2, MMP-3, MMP-13, and Granzyme B (Scott et al. 2000; Monfort et al. 2006; Boukpessi et al. 2008; Calabrese et al. 2011; Boivin et al. 2012). The concept that components released from the matrix act as DAMPs, activating the immune system during pathogen-mediated and sterile inflammation, has been presented in more detail in a recent review from our group (Moreth et al. 2012).

Early hints regarding the involvement of biglycan in the modulation of the inflammatory process were provided in several reports. The expression of biglycan was shown to be elevated in human fibroblasts from granulation tissue, suggesting its role in the development of chronic inflammatory lesions (Hakkinen et al. 1996). Biglycan levels were also increased in experimental pulmonary inflammation (Westergren-Thorsson et al. 1993) and bronchial mucosa of asthmatic patients (de Kluijver et al. 2005). In a murine model of unilateral ureteral obstruction (UUO), the upregulation of biglycan in the renal interstitium was followed by macrophage infiltration, indicating that biglycan might influence the initiation of renal inflammation (Schaefer et al. 2002). In mesangial cells from the renal glomerulus, biglycan expression is regulated by nitric oxide (NO) (Schaefer et al. 2003). As NO is a crucial proinflammatory mediator in glomerular kidney disease (Pfeilschifter 2002), involvement of biglycan in inflammatory processes is most likely.

### Biglycan: An Endogenous Ligand of Innate Immunity Receptors

**Figure 1.**
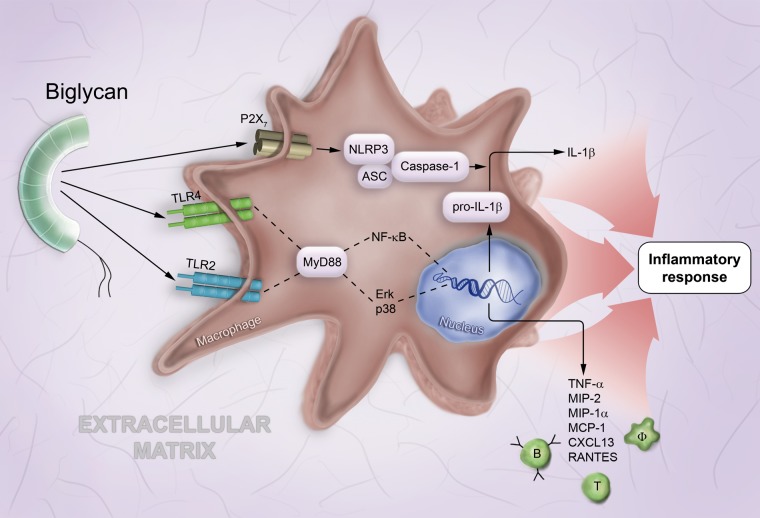
Biglycan-mediated proinflammatory signaling involves multireceptor crosstalk in macrophages. In macrophages (Φ), soluble biglycan interacts with TLR2 and TLR4 and triggers (via MyD88, NF-κB, Erk, and p38) the synthesis of proinflammatory cytokines, such as TNF-α and pro–IL-1β as well as various chemoattractants for macrophages and T and B lymphocytes, such as MIP-2, MIP-1α, MCP-1, CXCL13, and RANTES. By clustering TLR2/4 with the P2X7 purinergic receptor, biglycan induces the NLRP3/ASC inflammasome and caspase-1 activation with subsequent cleavage of pro–IL-1β and release of mature IL-1β. Abbreviations used in the figure: ASC, apoptosis-associated speck-like protein containing carboxy-terminal CARD; CXCL, C-X-C motif chemokine; Erk, extracellular signal-regulated kinase; IL, interleukin; MCP, monocyte chemoattractant protein; MIP, macrophage inflammatory protein; MyD88, myeloid differentiation primary response 88; NF-κB, nuclear factor kappa–light-chain enhancer of activated B cells; NLRP3, NLR family, pyrin domain containing; p38, mitogen-activated protein kinase p38; RANTES, regulated upon activation, normal T cell expressed and secreted; TNF, tumor necrosis factor; TLR, Toll-like receptor.

The very first indication that biglycan directly acts as a proinflammatory stimulus came from a study performed in murine primary peritoneal macrophages (Schaefer et al. 2005). This study provided evidence for biglycan-dependent signaling, describing the receptors involved, the downstream signaling events, and the inflammatory mediators subsequently generated (Schaefer et al. 2005). In its soluble form, biglycan is able to bind to both TLR2 and -4, triggering rapid activation of the mitogen-activated protein kinase p38, extracellular signal-regulated kinase (Erk), and nuclear factor kappa–light-chain enhancer of activated B cells (NF-κB) and consequently secretion of TNF-α. These signaling events were dependent on the MyD88 (myeloid differentiation primary response 88) gene (Fig. 1). Via TLR2/4-dependent signaling, biglycan triggers the synthesis of various chemoattractants for neutrophils and macrophages, such as MIP-1α (macrophage inflammatory protein–1α), MIP-2, MCP-1 (monocyte chemoattractant protein–1), and RANTES (regulated upon activation, normal T cell expressed and secreted) (Schaefer et al. 2005; Moreth et al. 2010) (Fig. 1). Subsequently, the newly attracted macrophages, being stimulated by proinflammatory cytokines, will in turn start to synthesize biglycan de novo, thereby enhancing the inflammatory response (Schaefer et al. 2005; Iozzo and Schaefer 2010; Schaefer 2010, 2011; Moreth et al. 2012). The role of biglycan in inflammation was also emphasized in vivo in biglycan-deficient mice, which showed longer survival correlated with a lower plasma level of TNF-α in lipopolysaccharide (LPS)-induced sepsis compared with wild-type animals (Schaefer et al. 2005).

Besides identifying biglycan as an ECM-derived DAMP, these studies have led to new concepts. First, biglycan has to be present in its soluble form, because when bound to the ECM, biglycan cannot act as a DAMP. Second, the magnitude of the biglycan signal can be ramped up rapidly by proteolytic liberation from ECM stores without the need for de novo synthesis. Third, both infiltrating macrophages, stimulated by proinflammatory cytokines and, at later time points, resident cells start to de novo synthesize biglycan at sites of injury or damage, in order to drive and shape the inflammatory response reaction over time. Fourth, by activating both the receptor for the Gram-negative (TLR4) and the Gram-positive (TLR2) bacterial response, biglycan, as a signal of tissue damage, acts as an amplifier for TLR-induced inflammation.

These initial findings were confirmed by several reports describing the coincidence of biglycan overexpression with enhanced inflammation and severe tissue injury (Wu et al. 2007; Leemans et al. 2009; Wang S et al. 2010; Schaefer 2011) in a TLR-dependent manner (Derbali et al. 2010; Moreth et al. 2010; Popovic et al. 2011). Furthermore, a number of other ECM components, such as decorin (Merline et al. 2011), hyaluronan (Termeer et al. 2002), versican (Kim et al. 2009), tenascin-C (Midwood et al. 2009), fibrinogen, and heparan sulfate fragments (Johnson et al. 2002), were identified as DAMPs alerting the innate immune system to the impending tissue damage. For further details, please refer to the review on the role of SLRPs in inflammation (Moreth et al. 2012).

### Biglycan: An Autonomous Trigger of the NLRP3 Inflammasome and IL-1β

Further studies indicated that proinflammatory effects of biglycan are mediated not only by its interaction with TLR2/4 but also by signaling through the NLR family, pyrin domain–containing 3 (NLRP3) inflammasome (Babelova et al. 2009). The NLRP3-inflammasome is a cytoplasmic protein complex, containing a Nod-like receptor (NLR), procaspase-1, and the adaptor molecule ASC (apoptosis-associated speck-like protein containing carboxy-terminal CARD). Activation of the inflammasome results in the maturation of caspase-1 with subsequent processing of pro–interleukin-1β (IL-1β) into mature IL-1β (Chen GY and Nunez 2010; Tschopp and Schroder 2010; Rathinam et al. 2012). Biglycan induces secretion of mature IL-1β, a proinflammatory cytokine important both in acute and chronic inflammation (Dinarello 2011), without any need for other costimulatory factors (Babelova et al. 2009). The NLRP3-dependent secretion of mature IL-1β usually requires two signals. The first signal, provided by ligands of TLRs or NOD2, activates NF-κB to synthesize pro–IL-1β and NLRP3. The second signal activates NLRP3/ASC and caspase-1 and leads to cleavage of pro–IL-1β (Chen GY and Nunez 2010; Tschopp and Schroder 2010). Surprisingly, biglycan alone is able to trigger both signals autonomously. By binding to and activating TLR2/4 signaling, biglycan induces the synthesis of pro–IL-1β and NLRP3. Based on co-immunoprecipitation data indicating the presence of the P2X7 purinergic receptor in the complex with TLR2/4, it is tempting to speculate that biglycan induces cooperativity between these receptors, thereby inducing NLRP3/ASC assembly, which finally leads to the activation of caspase-1 and release of mature IL-1β (Babelova et al. 2009) (Fig. 1). Both reactive oxygen species (ROS) and heat shock protein (HSP) 90 are involved in this process (Babelova et al. 2009). Further studies are needed to prove this concept and to exclude the independent engagement of individual receptors.

All studies so far showed that only intact biglycan is capable of triggering proinflammatory signaling both in macrophages and in dendritic cells (Schaefer et al. 2005; Babelova et al. 2009; Moreth et al. 2010; Popovic et al. 2011). Therefore, it appears that the combined ability of tandem LRRs and GAG side chains allows biglycan to interact with different cell surface receptors and their adaptor molecules, in order to cluster several types of receptors and orchestrate their signaling.

The biglycan-induced inflammasome activation was found to be of considerable relevance in vivo in two mouse models of sterile renal inflammation (UUO and Murphy Roths Large [MRL]/*lpr* lupus nephritis) and in the prototypic pathogen-mediated systemic inflammation of LPS-induced sepsis (Babelova et al. 2009; Moreth et al. 2010). It is conceivable that in sterile inflammatory diseases, soluble biglycan acts as an autonomous trigger of inflammation using receptor cooperativity between TLR2/TLR4 and the P2X7 receptor. In pathogen-mediated inflammation, biglycan appears to potentiate the inflammatory response via a second TLR, which is not involved in pathogen sensing (e.g., via a TLR2 in Gram-negative pathogen response).

In fact, recent reports indicated that biglycan and decorin are present in their soluble form in the extracellular space under sterile and pathogen-mediated inflammatory conditions (Moreth et al. 2010; Merline et al. 2011). The source of circulating biglycan still remains a matter of speculation. Probably both de novo synthesized and matrix-derived biglycan contribute to the circulating pool of this proteoglycan. De novo synthesis of biglycan can be triggered in various cell types by TGFβ (Border et al. 1990; Ungefroren and Krull 1996; Mozes et al. 1999). In macrophages, IL-6 and IL-1β have been shown to stimulate the synthesis of biglycan (Schaefer et al. 2002; Schaefer et al. 2005). It is conceivable that rapid generation of biglycan may exceed the capacity of the ECM to sequester this proteoglycan, causing some spillover of biglycan into the circulation. Furthermore, sequestered biglycan might be liberated from the ECM by proteolytic enzymes secreted from infiltrating or resident cells in response to tissue stress or damage.

### Biglycan Signaling: A Link between Innate and Adaptive Immunity

Recent studies established biglycan signaling as an important link between the innate and adaptive immune systems (Moreth et al. 2010; Popovic et al. 2011). In macrophages and dendritic cells, soluble biglycan induces the expression of CXCL13 (C-X-C motif chemokine 13) by signaling through TLR2/4 (Moreth et al. 2010). CXCL13 is the major chemoattractant for B cells and an important biomarker for disease activity of systemic lupus erythematosus (Fig. 1). In patients with lupus nephritis (LN) and in lupus-prone mice, enhanced plasma levels of biglycan correlate with the abundance of circulating CXCL13 and the extent of albuminuria. In lupus-prone mice, the knockout or overexpression of the biglycan gene was clearly associated with CXCL13 expression, number of B cells in the kidney, and organ damage and albuminuria (Moreth et al. 2010). It is conceivable that biglycan, by attracting B cells to non-lymphoid organs, promotes the development of tertiary lymphoid tissue and aggravation of the disease. Moreover, by overexpressing soluble biglycan in mice lacking TLR2 and TLR4, the first direct proof for the in vivo involvement of both TLRs in biglycan-mediated signaling was provided. Interestingly, soluble biglycan particularly facilitated the recruitment of B1 lymphocytes, which are involved in the early, T-cell–independent immune response (Moreth et al. 2010). Thus, these findings underline the role of biglycan as a potent inducer of inflammation, which can rapidly trigger autoantibody production without T-cell involvement.

However, biglycan-dependent regulation of adaptive immunity is not limited to the regulation of B lymphocytes. By signaling via TLR2/4, soluble biglycan also regulates the behavior of T lymphocytes. It induces the synthesis of RANTES, thereby recruiting T lymphocytes into the kidney (Moreth et al. 2010) (Fig. 1). In addition, by signaling through both TLRs and their adaptor molecules MyD88 and TRIF (TIR-domain-containing adaptor-inducing interferon β), biglycan plays a crucial role in MHC I– and MHC II–restricted T-cell cross-priming. Biglycan-mediated stimulation of TLR4 signaling is particularly important for MHC II-dependent, antigen-specific T-cell activation (Popovic et al. 2011). Accordingly, in a model of experimental autoimmune perimyocarditis (EAP), TLR4-dependent biglycan signaling amplified cardiomyocyte antigen presentation to prime T cells (Popovic et al. 2011). Beside the above-mentioned direct evidence for the impact of biglycan on bridging the innate and adaptive immune response, several further implications suggest an even wider impact of biglycan on both immune systems (Kikuchi et al. 2000; Kitaya and Yasuo 2009; Sjoberg et al. 2009). Details are summarized in a recent review (Moreth et al. 2012).

Taking all these findings into consideration, the aforementioned studies emphasize the prospect of biglycan as a therapeutic target for intervention in sterile and pathogen-mediated inflammation. Thus, further studies concerning the intricate interactions between biglycan and innate immune receptors would most likely reveal significant insights for the development of new drugs for the treatment of biglycan-mediated inflammatory diseases.

## Biglycan Signaling in Bone Formation

A role for biglycan in the growth of bone was suspected based on the observation that female patients with Turner syndrome, lacking the second X chromosome, have a shorter stature and abnormally low expression of biglycan, contrary to patients with supernumerary X chromosomes (Ameye and Young 2002). By the observation that biglycan-deficient mice display an osteoporosis-like phenotype, biglycan was discovered to be the first non-collageneous matrix component found in bone, being a regulator of bone formation and mass (Xu et al. 1998; Young et al. 2002). Identification of the underlying molecular mechanisms for developing this phenotype in biglycan-deficient mice has been of particular interest in the past years (Wadhwa et al. 2004; Young et al. 2006; Bi et al. 2007; Wadhwa et al. 2007; Embree et al. 2010; Berendsen et al. 2011).

**Figure 2.**
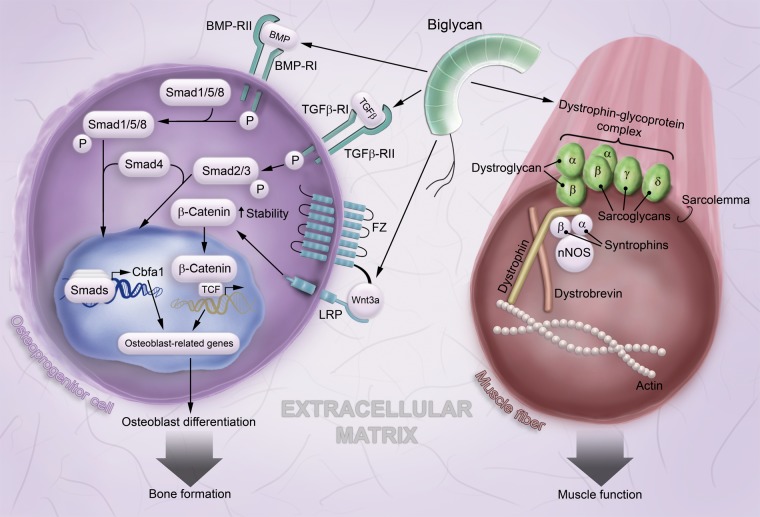
Network of biglycan signaling in osteoblast differentiation and stabilizing role of biglycan in skeletal muscle. In bone, biglycan stimulates the BMP/TGFβ pathways, leading to the transcription of osteoblast-related genes and osteoblast differentiation. By binding to the Wnt3a ligand and its receptor LRP6, biglycan potentiates the Wnt/β-catenin signaling pathway, thereby further contributing to osteoblast differentiation. In skeletal muscle, biglycan associates with the dystrophin-glycoprotein complex and contributes to its stability and to muscular integrity. For further details, refer to the text. Abbreviations used in the figure: BMP, bone morphogenic protein; BMP-R, bone morphogenic protein receptor; Cbfa1, core binding factor α1; FZ, Frizzled receptor; LRP, low-density lipoprotein receptor–related protein; nNOS, neuronal nitric oxide synthase; TCF, lymphoid enhancer binding factor/T-cell–specific factor; TGFβ, transforming growth factor β; TGFβ-R, transforming growth factor β receptor; Wnt, Wingless and Int.

It was shown that with increasing age, biglycan-deficient mice produce lower numbers of bone marrow-derived stromal cells (BMSC-osteogenic precursors). In addition, the response of BMSCs to TGFβ also becomes impaired, suggesting a possible role of BMP signaling in the development of this phenotype (Ameye and Young 2002; Chen XD et al. 2002). Indeed, biglycan was shown to modulate BMP4 (bone morphogenetic protein 4)-mediated osteoblast differentiation in murine calvarial cells by controlling Smad1 phosphorylation and Cbfa1 (core binding factor α1) expression (Chen XD et al. 2004) (Fig. 2). Another study also confirmed the role of biglycan in osteoblast differentiation and subsequent matrix mineralization through the BMP4 signaling pathway (Parisuthiman et al. 2005). An opposite effect of biglycan in BMP4 signaling was shown in the context of embryonic development. Microinjection of biglycan mRNA into *Xenopus* embryos inhibits BMP4 activity and affects embryonic development. At the molecular level, biglycan binds to BMP4 and chordin, a negative regulator of BMP4, increasing the binding efficiency between the aforementioned proteins, thereby blocking BMP4 activity (Moreno et al. 2005). Besides BMP4, in vitro binding assays showed that biglycan interacts with other BMPs, such as BMP2 and 6. Biglycan is able to directly bind BMP2 and its receptor, ALK6 (also known as BMP-RIB), to stimulate BMP2-dependent osteoblast differentiation (Moreno et al. 2005). Moreover, de-glycanation of biglycan increases its positive effect on BMP2 signaling and function (Miguez et al. 2011).

However, the signaling role of biglycan in bone is not restricted to interactions with BMPs. Biglycan is also able to activate the canonical Wnt/β-catenin signaling (Fig. 2). The activation of the Wnt/β-catenin pathway involves the binding of Wnt ligand to the Frizzled receptor (FZ) and its coreceptor, LRP6, increasing the stability of β-catenin in the cytosol and promoting its translocation into the nucleus. Consequently, β-catenin activates LEF1/TCF (lymphoid enhancer-binding factor/T-cell-specific factor)-related gene transcription (Berendsen et al. 2011) (Fig. 2). Biglycan directly binds to the Wnt ligand and LRP6 through its protein core. The lack of biglycan led to impaired Wnt-induced LRP6 phosphorylation and LEF1/TCF-mediated transcriptional activity in calvarial cells. The same study showed that in vivo, biglycan regulates WISP1 expression during bone formation in a fracture-healing model (Berendsen et al. 2011). Nevertheless, further studies are needed to elucidate the molecular mechanism, through which biglycan regulates the Wnt pathway in more detail.

Based on findings that biglycan directly binds to WISP1 protein (Desnoyers et al. 2001) and that WISP1 mRNA colocalizes with biglycan during mineralization in vivo (French et al. 2004; Inkson et al. 2009), it has been suggested that this interaction plays a role in the differentiation and proliferation of osteogenic cells. Briefly, the matrix component biglycan stimulates the bone formation process through a dual-signaling mechanism: BMP/TGFβ signaling and the canonical Wnt/β–catenin-induced pathway (Fig. 2). Therefore, biglycan might be a promising drug target in bone-related diseases caused by defects in these signaling pathways.

## Muscular Dystrophies: Regulatory Mechanisms of Biglycan and Novel Therapeutic Options

In early studies, biglycan was shown to be expressed in muscle tissue (Bianco et al. 1990; Bosse et al. 1993), but it took almost a decade until a study was published on the role of biglycan in muscle, which demonstrated that biglycan binds to dystroglycan and that its expression level is increased in the muscle of *mdx* mice, a model of Duchenne muscular dystrophy (DMD) (Bowe et al. 2000). Later, increased levels of biglycan were also found in skeletal muscle of DMD patients (Zanotti et al. 2005; Fadic et al. 2006).

DMD is a lethal X-linked recessive disorder caused by mutations in the dystrophin gene that lead to a shift in the reading frame and an early stop codon, causing the loss or reduction in the synthesis of the dystrophin protein, which finally prevents the assembly of the dystrophin-glycoprotein complex (DGC) (Ameen and Robson 2010). The DGC is a large protein complex including both cytoplasmic components (dystrophin, syntrophins, dystrobrevins, and neuronal nitric synthase [nNOS]) and transmembrane components (dystroglycan and sarcoglycans) (Brandan et al. 2008). Lack of this complex renders the muscle fibers extremely vulnerable to damage, which occurs during muscle contraction. This gives rise to repeated cycles of muscle damage and regeneration as the organism tries to cope with this condition but finally results in depletion of regenerative myogenic cells and loss of regenerative ability (Brussee et al. 1997; Luz et al. 2002). The most commonly used model of DMD is the *mdx* mouse, which has a nonsense mutation in exon 23 of the dystrophin gene (Zhou and Lu 2010).

Biglycan has been shown to bind to several components of the DGC: α-dystroglycan (Bowe et al. 2000) as well as α- and γ-sarcoglycan (Rafii et al. 2006). Although O-linked glycosylation of α-dystroglycan is needed for its interaction with other proteoglycans (agrin, laminin, perlecan), the interaction with biglycan occurs between the GAG chains of biglycan and the carboxy-terminal one-third of α-dystroglycan, indicating that α-dystroglycan is able to bind two molecules of biglycan simultaneously (Bowe et al. 2000). By contrast, biglycan interacts with α- and γ-sarcoglycan but not with β- or δ-sarcoglycan through distinct parts of its protein core (Rafii et al. 2006).

These interactions with three distinct components of the DGC (Fig. 2) led to the idea that biglycan is associated with this complex. As proof of this concept, biglycan was shown to co-immunoprecipitate with α-, β-, γ-sarcoglycan as well as with dystrophin, even though it did not directly bind to dystrophin or β-sarcoglycan. Therefore, co-immunoprecipitation with these two proteins was concluded to be a result of biglycan association to the assembled DGC. Moreover, biglycan selectively regulates the expression of α- and γ-sarcoglycan, with expression of these two proteins being reduced in muscle from young biglycan-null mice but not adults (Rafii et al. 2006).

In addition, a further study on biglycan-null mice showed that biglycan also regulates the expression and sarcolemmal localization of other DGC components: dystrobrevin, synthrophin, and nNOS. Biglycan-null mice have a mildly dystrophic phenotype and present several defects in the localization of DGC components. α-Dystrobrevin-1 and -2 have a selective reduction in their localization at the sarcolemma. On the other hand, nNOS is also decreased transcriptionally in null mice. Different types of synthrophins are affected differently by a lack of biglycan, with the largest effect on β1-syntrophin. Remarkably, sarcolemmal localization of these components in biglycan-null mice can be restored by the injection of purified biglycan core protein into muscle (Mercado et al. 2006). Thus, biglycan seems to be capable of regulating multiple components of the DGC (Fig. 2) and shows some potential as a therapeutic agent in the treatment of muscle dystrophy.

In a study performed in *mdx* mice, a therapeutic role for biglycan has indeed been proven (Amenta et al. 2011). Absence of biglycan was shown to decrease the sarcolemmal expression of utrophin, the autosomal homologue of dystrophin, whereas injection of recombinant human biglycan in biglycan-null mice increased the expression of utrophin. A similar effect was shown in *mdx* mice, where injection of biglycan could increase the expression of utrophin in muscle 2.5-fold, associated with an increase in γ-sarcoglycan, β2-syntrophin, and nNOS levels at the sarcolemma. Furthermore, injection of biglycan in *mdx* mice ameliorated dystrophic symptoms, depending on the presence of utrophin (Amenta et al. 2011). Importantly, the therapeutic effects of biglycan in an experimental model of DMD are mediated by the protein core. Therefore, proinflammatory effects of biglycan are not to be expected, as intact biglycan encompassing the GAG side chains is needed for signaling through TLR2 and TLR4.

Besides its role in the assembly of the DGC, biglycan has also been shown to be upregulated in regenerating skeletal muscle, although the presence of biglycan is not essential for muscle regeneration and is possibly compensated for by decorin in biglycan-null mice (Casar et al. 2004). Through its binding to TGFβ, biglycan could also potentially modulate processes such as proliferation, migration, and differentiation (Brandan et al. 2008). In addition, biglycan expression was recently localized to the neuromuscular junction, whereas mature biglycan-null mice have abnormal neuromuscular synapses and have a reduced synaptic expression of the receptor tyrosine kinase MuSK. Consequently, it was shown that biglycan serves as a ligand for MuSK, regulates agrin-induced MuSK phosphorylation, and is necessary for stabilizing agrin-induced acetylcholine receptor clusters. Taken together, these results suggest that biglycan also plays an important role in the stability of neuromuscular synapses (Amenta et al. 2012).

## Future Perspectives

Research over the past few years has resulted in considerable progress in our understanding of the biology of biglycan. Particular attention was focused on the interaction between biglycan and various cell surface binding partners, some of them being signaling receptors. The initial concept that soluble biglycan acts a signaling molecule became much more complex when new data showed that biglycan is capable of clustering different types of receptors on the cell surface, thereby orchestrating their downstream signaling events.

Current knowledge on biglycan in inflammation, bone development, and muscular dystrophy summarized in this review suggests that besides general effects of this proteoglycan acting as a danger signal in matrix stress or injury, other effects are strongly tissue specific. There is good evidence demonstrating the tissue-specific effects of biglycan in muscular dystrophy and in bone formation. However, it is conceivable that in contrast to muscle-specific association of biglycan with the dystrophin-glycoprotein complex, its interactions with BMP/TGFβ and Wnt/β-catenin pathways may play an important role not only in bone formation but also in fibrotic disorders. Thus, new studies in various tissues under physiological and pathological conditions should expand the current knowledge.

The challenges ahead lie in transferring basic research on biglycan into the clinic. Both in sterile and pathogen-induced inflammatory conditions, the interaction of biglycan with innate immunity receptors might represent a promising target for the development of new anti-inflammatory therapies. Possible strategies for neutralizing the proinflammatory effects of biglycan might include neutralizing antibodies, truncated molecules, or chemically synthesized small molecules. On the other hand, biglycan by itself could serve as a promising treatment in some skeletal muscular dystrophies. A more detailed understanding of the physicochemical and structural properties of the binding sites through which biglycan interacts with its various binding partners will be a critical component of this effort.
